# Uric Acid Accumulation in Gout

**Published:** 1903-08-01

**Authors:** 


### URIC ACID ACCUMULATION IN GOUT.

Although there are few maladies which have received more attention at the hands of clinicians than gout, yet it can hardly be claimed that our knowledge of the condition has advanced materially in recent years either with respect to its pathology or its successful treatment. The manifestations which have proved most alluring to investigators are the accumulation of uric acid in the blood, and the formation of deposits of crystalline urates in certain parts of the body. Taking the former of these, it is obvious that the excess of uric acid in the blood of a gouty patient may be due, theoretically, to one or more of three causes—viz. : (1) excessive formation ; (2) deficient elimination ; (3) deficient destruction. Dr. Croftan,[1] of Chicago, has recently carried out a large number of laboratory experiments which have a bearing on the last of these possibilities. He first set to work to discover, if possible, whether or not uric acid is normally destroyed when circulating in the blood. Others have worked at this point, but Dr. Croftan was not satisfied that every source of fallacy had been excluded and he therefore devised a very elaborate technique which he thought would obviate the likelihood of wrong conclusions. His experiments were carried out on rabbits, and he found that in these animals between 80 and 90 per cent. of uric acid injected intravenously undergoes disassimilation within one to two hours, that none of it is retained, and that it is not excreted into the bowel. Reasoning from analogy and from the fact that isolated human organs have the power of destroying uric acid, it is quite a fair assumption, Dr. Croftan thinks, that the same process occurs with circulating uric acid in the human. For many reasons it is impossible to carry out injection experiments on the human and the only experimental path that is open is the investigation of the power possessed by isolated human organs of destroying uric acid. This leads to three inquiries, namely : Where is uric acid destroyed? How is uric acid destroyed? What becomes of the uric acid? As the result of a large number of experiments carried out with the isolated organs of several species of animals Dr. Croftan found that in carnivora the liver was by much the most potent organ in destroying uric acid. In the herbivora, on the other hand, the kidneys are the only organs found to possess the function in a high degree. In omnivora, including man and the pig, both the kidneys and liver possess a high power of destroying uric acid ; in man the kidneys are somewhat more potent than the liver in this respect. These results denote weight for weight the power of uric acid destruction which each organ is capable of, but if the relative bulk of each tissue is considered the muscles would appear to destroy as much, if not more, uric acid than the liver. The actual process of uric acid destruction appears to be one of oxidation, and the active agent, Dr. Croftan thinks, is an albumose which is assisted by a nucleo-proteid which functions as an oxygen carrier. In studying the effect of various salt solutions on the catalytic action of the nucleo-proteid it was found that certain salts (*e.g.* nitrates and cyanates) greatly diminished the power of the nucleo-proteid to liberate oxygen from a given quantity of hydrogen peroxide, whereas certain other salts including salicylates and dilute alkalies greatly increased this power. With regard to the third part of the inquiry no satisfactory conclusions could be formed as to the substances which result from the breaking down of the uric acid. On the whole these experiments lend support to the renal theory of gout. As the majority of cases of goutiness begin with renal insufficiency, and as nearly all advanced cases show typical kidney lesions, the connection between perversions of the uric acid destroying powers of the kidneys and the development of uratic deposits is evident. The fact that in lead poisoning granular nephritis precedes the development of gouty symptoms lends additional support to the view. Moreover the power possessed by human muscle to destroy uric acid may explain why exercise often relieves uratic symptoms ; the increased flow of blood carrying uric acid through the muscles would lead to greater uric acid destruction, and would therefore be beneficial to the patient.

[1] Medical Record, July 4.

